# Giant Atonic Bladder (4000 mL) in the Postpartum Period: A Case Report

**DOI:** 10.1155/criu/1448191

**Published:** 2025-10-08

**Authors:** Nona Sabeti, Leila Pourali, Mahdieh Mottaghi, Atiyeh Vatanchi

**Affiliations:** ^1^Supporting the Family and the Youth of Population Research Core, Department of Obstetrics and Gynecology, Faculty of Medicine, Mashhad University of Medical Sciences, Mashhad, Iran; ^2^Clinical Research Development Unit, Ghaem Hospital, Mashhad University of Medical Sciences, Mashhad, Iran

**Keywords:** intermittent catheterization, postpartum urinary retention, voiding dysfunction

## Abstract

**Background and Aim:**

Postpartum urinary retention (PUR) is a well-recognized complication of childbirth. The prolonged duration and exceptionally large residual bladder volume of 4000 mL observed in this case, despite the patient's report of spontaneous voiding on the first postpartum day, is rare.

**Case Report:**

A 21-year-old primiparous woman presented on Postpartum Day 16 with abdominal distension. She reported no urinary symptoms. Her condition had previously been misattributed to postpartum infection during an earlier admission. She was diagnosed with covert PUR, and catheterization drained 4000 mL of urine. However, after 4 days of catheterization, the patient remained unable to void spontaneously. She was then managed with clean intermittent catheterization (CIC) for 2 weeks, and urinary tract function gradually recovered.

**Conclusion:**

This case report stands out due to the extraordinary bladder volume and protracted course, providing a unique perspective on the spectrum of PUR severity. While routine postpartum discharge protocols rely on spontaneous voiding, this case emphasizes the importance of thorough subjective assessment of lower urinary tract symptoms (LUTSs) for early recognition of PUR to prevent such extreme presentations.

## 1. Introduction

Postpartum urinary retention (PUR) is a significant condition that may cause long-lasting consequences [1]. PUR prevalence varies widely, with studies reporting rates from 0.05% to 47%, influenced by different definitions [2, 3]. Based on clinical presentation, PUR is classified as overt (symptomatic), covert, and persistent (protracted) [4]. Overt PUR is described as the inability to void within 6 hours after vaginal delivery. Patients with covert PUR can micturate spontaneously; however, there is a postvoid residual (PVR) bladder volume greater than 150 mL [5, 6]. The risk of underdiagnosis is high when patients present with nonspecific symptoms [7].

While PUR is most commonly observed within the first 6 h postpartum [8], 0.05%–0.18% of the general obstetric population develops protracted PUR, described as the inability to void spontaneously or a residual volume more than 200 mL beyond the third postpartum day [9–12].

Giant atonic bladder is a rare clinical condition with no clear consensus on diagnostic criteria [13]. It typically progresses gradually and remains mostly asymptomatic despite substantial urinary retention exceeding 1000 mL [13]. Previous studies have reported cases of giant atonic bladder with extreme bladder volumes of 6000, 10,500, and even 11,000 mL, often associated with chronic conditions such as neurogenic bladder dysfunction or obstructive uropathy [13–15]. However, a residual bladder volume of 4 L in the postpartum setting is exceptionally rare and, to the best of current knowledge, has not been previously documented.

Herein, we report a case that initially presented as covert PUR and subsequently progressed to persistent PUR with a volume of 4 L. The originality of this case lies in the unprecedented volume and duration of PUR.

## 2. Case Presentation

A 21-year-old primiparous female presented on Day 16 postpartum with abdominal distention and lower abdominal pain. She had delivered a 3450-g male infant with an Apgar score of 9–10 at 39 weeks' gestation via vaginal delivery with a mediolateral episiotomy. Delivery was complicated by a third-degree perineal laceration, which was repaired immediately postpartum. The duration of labor was 525 min, with the second stage lasting 95 min. The patient had no significant past medical history and was discharged on the first postpartum day after reporting spontaneous voiding.

One week prior to her current admission, the patient had presented with similar complaints. She was diagnosed with a postpartum perineal infection due to an episiotomy wound infection. She received intravenous (iv) ceftriaxone 1 g twice daily and iv clindamycin 900 mg three times daily for 1 day. Due to clinical deterioration, including persistent high-grade fever ranging between 38.2°C and 39.4°C, the patient was diagnosed with postpartum fever, and the antibiotic regimen was changed to iv meropenem 1 g three times daily and iv vancomycin 500 mg three times daily for 3 days.

**Figure 1:**
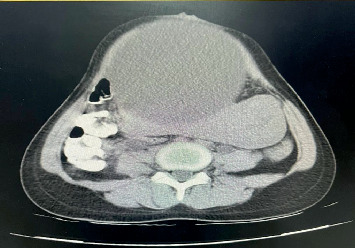
Abdominal computerized tomography (CT) scan showing an enlarged bladder and displaced uterus.

An abdominopelvic computed tomography (CT) scan with and without contrast was performed to assess the probable source of infection. The scan demonstrated a distended bladder measuring 19 × 20 × 17 cm (Figure 1) and a displaced uterus. Consequently, bladder catheterization was recommended; however, the patient left the hospital against medical advice.

**Figure 2:**
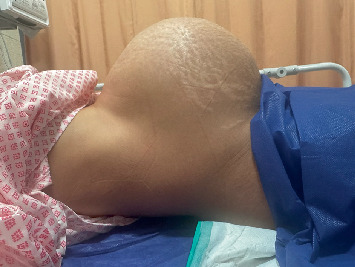
Abdominal distension on Postpartum Day 17, with fundal height consistent with term gestation, prior to urinary catheterization.

Upon her readmission, her vital signs were stable, with no fever or tachycardia. Abdominal examination revealed marked abdominal distension, with a fundal height corresponding to a term gestation (Figure 2), along with tenderness and guarding in the suprapubic region. Digital vaginal examination showed bulging of the anterior vaginal wall and bladder into the vaginal canal. Speculum examination revealed no foul-smelling discharge or any signs suggestive of postpartum infection. The patient denied any history of urinary problems. However, a detailed history regarding lower urinary tract symptoms (LUTSs) revealed difficulty initiating urination, an intermittent stream, the need for suprapubic pressure to void, and a sensation of incomplete emptying.

The laboratory tests were as follows: hemoglobin 11.8 g/dL, hematocrit 35.1%, white blood cells 11,200 cells/mm^3^, thrombocytes 168,000 cells/mm^3^, and plasma creatinine 0.8 mg/dL. Given the clinical suspicion of persistent urinary retention, an 18-F Foley catheter was inserted, which drained 4000 mL of urine over 6 h with intermittent clamping.

**Figure 3:**
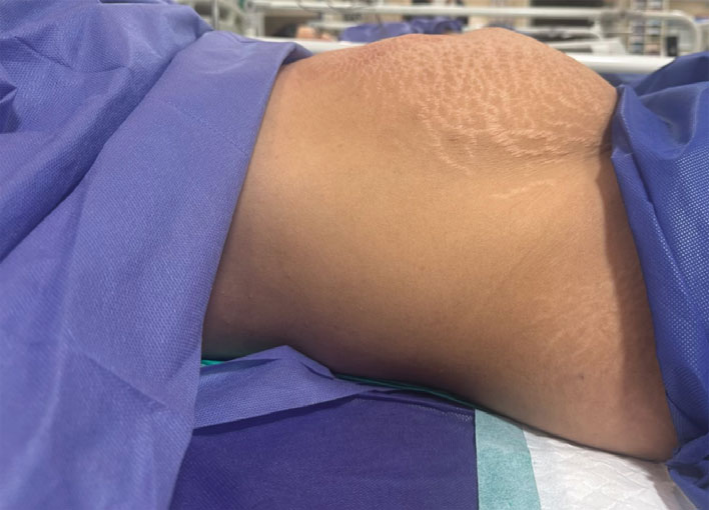
Abdominal distension improved after urinary catheterization and drainage of 4000 mL of urine.

Following bladder decompression by catheterization, her abdominal distension resolved, and the abdomen became nontender on examination (Figure 3). The urinary catheter remained for 4 days. On Postpartum Day 20, the patient was discharged on clean intermittent catheterization (CIC) every 4–6 h to help complete bladder emptying and oral bethanechol 10 mg daily. After 2 weeks of CIC, bladder function gradually recovered.

At the 1-month follow-up, although urodynamic studies were not conducted, the patient demonstrated normal voiding patterns, with no need for catheterization, no episodes of incontinence or dribbling, and no straining or hesitancy. A repeat ultrasound confirmed a PVR volume of 40 mL, which is within normal limits.

## 3. Discussion and Conclusions

We presented a case of PUR with both covert and protracted symptoms, initially misdiagnosed as a puerperal infection. Delayed diagnosis of PUR can have significant clinical consequences, such as extreme bladder distension or spontaneous rupture [16].

Notably, the prevalence of covert PUR decreases over the postpartum period, with 19% at 6 h, 15% at 24 h, 11% at 1 day, 7% at 2 days, 8% at 3 days, and only 0.1% beyond 4 days postpartum [8], underscoring the rarity of our case. A previous study reported a case of a 23-year-old primiparous woman with persistent urinary retention lasting over 30 days postpartum; however, specific volume was not detailed [17].

In this case, primiparity, mediolateral episiotomy, and third-degree perineal laceration were notable risk factors for PUR. A systematic review conducted in 2024, encompassing 27 studies, identified the risk of PUR after vaginal delivery as follows: primiparity (OR = 2.36, 95%CI = 1.64‐3.38), mediolateral episiotomy (OR = 3.65, 95%CI = 1.70‐7.83), and severe perineal tear (OR = 3.21, 95%CI = 1.84‐5.61) [18]. Furthermore, another study indicated that episiotomy is associated with an elevated risk of PVR bladder volume of more than 500 mL (OR = 3.72, 95%CI = 1.71‐8.08) [3].

Our patient reported spontaneous urination on the first postpartum day. She later experienced episodes of urinary dribbling and the need for suprapubic pressure. However, on her first admission, she did not complain about urinary symptoms and presented with abdominal pain. Routine postpartum discharge protocols typically include inquiries about spontaneous voiding, excluding overt PUR. However, taking a more detailed history on LUTS is necessary to identify whether the amount of urination is smaller than normal or none. Furthermore, a focused physical examination including bladder palpation and vaginal inspection is crucial in detecting signs of urinary retention, such as a vesical globe or bulging of the bladder into the vaginal canal, which was later observed in our patient [19]. Imaging modalities should support, not replace, the critical role of comprehensive history-taking and physical examination for early detection of PUR [20].

LUTS is a broad term encompassing a range of urinary complaints, typically categorized into (1) storage symptoms such as urinary frequency, urgency, and nocturia and (2) voiding symptoms, including a weak or intermittent stream, hesitancy, and straining [21]. The questionnaire for subjective assessment of LUTS has been validated in previous observational studies [22]. Thus, a detailed history-taking regarding LUTS and physical examination could be implemented for the screening of covert PUR.

First-line management of urinary retention typically involves bladder drainage via catheterization. Complications following decompression include hypotension, hematuria, and postobstructive diuresis [23]. Previous research has evaluated whether the rate of decompression, rapid versus gradual, affects the likelihood of associated complications. A 2022 meta-analysis indicated that while hematuria was relatively more prevalent with rapid bladder decompression, the difference was not statistically significant (RR = 0.91; 95%CI = 0.6‐1.3; p  = 0.6) [24]. In a case-control study of 62 patients with BPH-related acute urinary retention, rapid decompression led to a mean blood pressure drop of 15 mmHg compared to 10 mmHg with gradual decompression (*p* > 0.05) [25]. Although rapid decompression may be associated with slightly greater physiological changes, current evidence supports its safety and efficacy.

It is noteworthy that most previous studies have been conducted on patients with typical volumes of urinary retention. The majority of patients demonstrated residual bladder volumes in the range of 1000–1500 mL at the time of diagnosis [26]. Previous case reports involving massive bladder distention employed gradual bladder decompression [13, 15].

However, one study reported a 75-year-old man with BPH-related urinary retention, managed with rapid catheterization, which resulted in the drainage of 5.9 L of urine. The patient subsequently developed hypovolemic shock due to rapid diuresis and experienced severe, persistent hematuria requiring four units of packed red blood cells, multiple bladder irrigations, repeated cystoscopic interventions, and intravesical antifibrinolytics for hemostasis [26]. This case underscores the potential for life-threatening complications following rapid decompression of a severely distended bladder, emphasizing the need for individualized management in extreme retention scenarios.

Persistent PUR in our patient was managed with an indwelling catheter for 4 days. Current recommendations comparing CIC with indwelling catheterization for initial bladder decompression in the management of persistent PUR are limited. A multicenter randomized controlled trial compared CIC every 6 h (*n* = 73) with 24-h indwelling catheterization (*n* = 74) in women diagnosed with PUR within 6 h postpartum. CIC led to significantly faster resolution (10.2 ± 11.8 vs. 26.5 ± 9.0 h; *p* < 0.001), with 99% resolving by 24 h versus 91% in the indwelling group (*p* = 0.04), and was associated with higher patient satisfaction (*p* < 0.01) [27]. On the other hand, physiological conditions such as increased fluid intake due to lactation, vulvar edema, and postpartum diuresis complicate early initiation of CIC [4]. An expert review supports initial catheterization of patients with PUR for 5–7 days [4]. In the present case, the indwelling catheter remained during hospitalization, and the patient was discharged on CIC. The patient remained under inpatient observation for 4 days to ensure no recurrence of bladder overdistension, to monitor for potential complications related to bladder decompression, and to confirm adequate patient education and adherence to CIC prior to discharge.

In our case, outpatient management after discharge included CIC for 14 days every 6 h. It is recommended that the frequency of CIC should be guided by the volume of PVR urine. For patients with PVR volumes greater than 400 mL, catheterization is advised four to six times per day, with the frequency gradually reduced based on PVR measurements. This approach is aimed at preventing bladder overdistention while minimizing the burden of catheterization and should be individualized based on the patient's clinical progress [4].

One limitation of our study was the lack of long-term follow-up, which limited our ability to comprehensively assess late-onset urinary complications. Long-term follow-up studies indicated that women undergoing complex obstetric surgeries may develop LUTS months after delivery, underscoring the importance of prolonged follow-ups in high-risk patients [28]. Further longitudinal research is warranted to elucidate the potential long-term outcomes associated with severe protracted PUR.

In conclusion, our recommended management steps for patients presenting with large-volume retention (e.g., > 2000 mL) include that bladder decompression via a Foley catheter is indicated, with gradual drainage over several hours (e.g., intermittent clamping over 4–6 h) to minimize the complications. In cases of persistent PUR, where spontaneous voiding does not resume after 3–4 days, an indwelling catheter may be left in place for up to 5–7 days. Following this, CIC should be initiated, typically every 4–6 h, with adjustments based on PVR measurements, catheterization five to six times daily if PVR is over 400 mL, three to four times daily if between 200 and 400 mL, and reduced frequency as volumes decline. PUR should be considered in the differential diagnosis of postpartum women presenting with abdominal distention, even in the absence of overt urinary symptoms.

## Data Availability

The data that support the findings of this study are available from the corresponding author upon reasonable request.

## Nomenclature

CIC: clean intermittent catheterization
CT scan: computed tomography scan
iv: intravenous
LUTS: lower urinary tract symptoms
PUR: postpartum urinary retention
